# Proprotein Convertase Subtilisin/Kexin 9 as a Modifier of Lipid Metabolism in Atherosclerosis

**DOI:** 10.3390/biomedicines11020503

**Published:** 2023-02-09

**Authors:** Anastasia V. Poznyak, Vasily N. Sukhorukov, Ilya I. Eremin, Irina I. Nadelyaeva, Nikita A. Gutyrchik, Alexander N. Orekhov

**Affiliations:** 1 Institute for Atherosclerosis Research, Osennyaya 4-1-207, 121609 Moscow, Russia; 2 Institute of General Pathology and Pathophysiology, Russian Academy of Medical Sciences, 125315 Moscow, Russia; 3 Petrovsky National Research Centre of Surgery, 2, Abrikosovsky Lane, 119991 Moscow, Russia

**Keywords:** PCSK9, atherosclerosis, cardiovascular disease, CVD, lipids, lipid metabolism

## Abstract

Despite being the most common treatment strategy in the management of atherosclerosis and subsequent cardiovascular disease, classical statin therapy has certain disadvantages, including numerous side effects. In addition, a regimen with daily administration of the drug is hard to comply with. Thus, there is a need for modern and more efficient therapeutic strategies in CVD treatment. There is extensive evidence indicating that PCSK9 promotes atherogenesis through a variety of mechanisms. Thus, new treatment methods can be developed that prevent or alleviate atherosclerotic cardiovascular disease by targeting PCSK9. Comprehensive understanding of its atherogenic properties is a necessary precondition for the establishment of new therapeutic strategies. In this review, we will summarize the available data on the role of PCSK9 in the development and progression of atherosclerosis. In the last section, we will consider existing PCSK9 inhibitors.

## 1. PCSK9 Gene and Protein

PCSK9, sometimes referred to as neural-apoptosis-regulated convertase 1 (NARC-1), is a member of proprotein convertases (PCs). PCSK9 is predominantly generated and expressed in the liver. However, its secretion may also be observed in intestine, lung, kidney, central nervous system, as well as in endothelial and smooth muscle cells (SMC) [1].

The PCSK9 gene is located on the short arm of chromosome 1 (chr1p32.3). It includes 12 exons and 11 introns and has a length of 22 kb. There are numerous transcription factors that determine the gene’s expression, including sterol-response element binding proteins 1 and 2 (SREBP1 and SREBP2), which have been extensively studied [2]. For example, in vitro and in vivo studies have demonstrated PCSK9 gene expression by SREBP2 can be increased by elevated sterol levels after feeding. Similarly, higher postprandial insulin levels promote PCSK9 mRNA synthesis as a result of SREBP1 activation [3,4]. Still, clinical evidence indicates that insulin-mediated PCSK9 gene expression has little or no clinical relevance. The expression of the gene can also be affected by peroxisome proliferator-activated receptor γ and α (PPAR and PPARα) transcription factors. While PPARα decreases its expression, PPARγ promotes the hepatic gene expression of proprotein convertase. Finally, PCSK9 gene expression can be regulated by sirtuin 1 and 6 (SIRT1 and SIRT6), which can silence the PCSK9 gene [5].

The PCSK9 gene encodes for pro-PCSK9, a protein with a molecular weight of 72 kDa which consists of 692 amino acids. Similarly to LDLR, which it mainly targets, pro-PCSK9 is synthesized in the endoplasmic reticulum (ER). The protein consists of five segments: a signal peptide (aa 1–30); an N- terminal prodomain (aa 31–152) that is cleaved in the ER; a catalytic domain (aa 153–404) containing the active sites Asp186, His226, and Ser386; a small hinge region of 18 amino acids region (amino acids 405–454) connecting the catalytic domain to the C-terminal cys-his-rich domain (CHRD) (aa 440–692). Pro-PCSK9 maturation requires post-translational modifications. For instance, the N-terminal prodomain of pro-PCSK9 at the Val-Phe-Aln-Gln152Ser-IIe- Pro (VFAQ152SIP) site is autocatalytically cleaved in the ER. However, it remains non-covalently bound to the rest of the mature protein, promoting PCSK9 folding and blocking its catalytic domain [6].

Following the synthesis and cleavage, PCSK9 is transported to the Golgi apparatus. The transfer from the ER to the Golgi apparatus is mediated by transport protein A belonging to the COPII coat complex (Sec24A). Similarly, the association between sortilin 1 (SORT1) and PCSK9 in the trans-Golgi is essential for the inactive protease expression through the blood [7].

One of the central components of liver oncogenesis is lipid metabolism. Therefore, PCSK9, of course, is in the spotlight here as well, in addition to standard LDLR binding and targeting lysosome destruction. PCSK9 promotes the degradation of other lipoprotein receptors such as VLDLR, ApoER2, CD36, and LRP1. This makes PCSK9 an attractive target for developing cancer therapies [8].

It has been shown that, in Alzheimer’s disease, elevated levels of PCSK9 are present in the cerebrospinal fluid, which may further influence brain cholesterol metabolism in neurons and astrocytes, as well as exacerbate Aβ-mediated neurotoxicity. This testifies to the attractiveness of PCSK9 as a therapeutic target. Consistent with this suggestion, in animal models of high-fat diet-induced cognitive impairment, administration of a PCSK9 inhibitor was associated with decreased Aβ1-42 production, microglial activation, hippocampal apoptosis, and lower cognitive decline. However, the results of various studies are not fully consistent with each other. Recently, these results have been confirmed in a specific mouse model of Alzheimer’s disease. In these mice, monoclonal antibody inhibition of PCSK9 resulted in an improvement in hippocampal-dependent memory functions, which occurred by suppressing LRP1-mediated Aβ clearance. The authors of the study suggested that this is due to the peripheral effect of PCSK9, given the inability of anti-PCSK9 antibodies to cross the BBB. In this context, our data on the direct effects of PCSK9 on brain cells are valuable in that they may pave the way for the development of new anti-PCSK9 strategies based, for example, on small lipophilic molecules that enter the CNS and act directly on astrocytes and neurons. This new pharmacological approach, focused on the lipid hypothesis of AD pathogenesis, will open up new perspectives for AD, for which there are currently no available therapies [9].

## 2. PCSK9 Role in Lipid Homeostasis

It is well-known that PCSK9 is involved in cholesterol metabolism. The most-studied mechanism through which PCSK9 influences the amount of cholesterol is by promoting lysosomal LDLR degradation. LDLR in its turn, is involved in PCSK9 homeostasis as it mediates its clearance from plasma, irrespective of the PCSK9 tissue origin [10].

**Figure 1:**
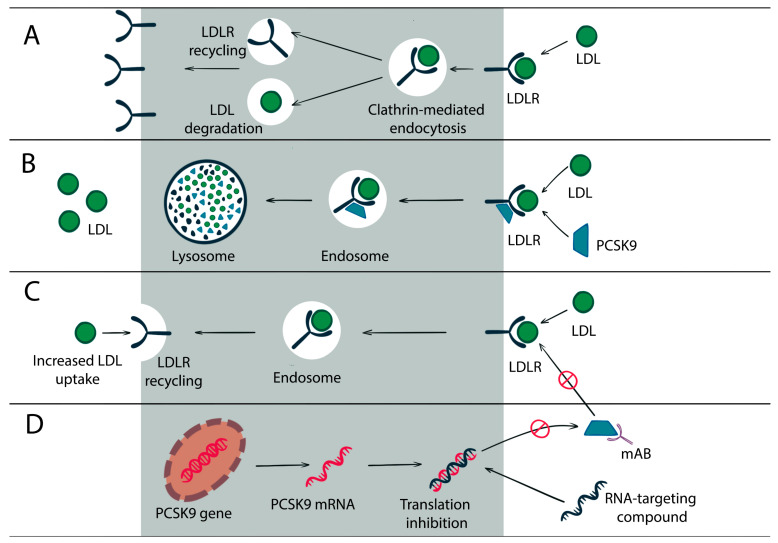
PCSK9 and LDLR. Panel (**A**): the LDLR–LDL complex internalization into endosomes through a clathrin-dependent mechanism. After that, LDLR is recycled to the cell surface. Panel (**B**): in the presence of PCSK9, the LDLR–LDL complex is internalized into endosomes, and then all parts are degraded in lysosome. Panel (**C**): in the presence of mAB, LDLR is internalized and then recycled to the cell surface. Panel (**D**): the RNA-targeting compound binds to PCSK9 mRNA and prevents its translation.

LDLR enters the cell in association with LDL-C, unless it had been bound to PCSK9. It is detached from the LDL-C in the endosomal space and recycled to the surface of the cell, while LDL-C is transferred to the lysosomes, where it is degraded. If LDLR is bound to PCSK9, it is degraded via endocytosis. PCSK binding to LDLR can happen in two phases: rapid-phase binding (5–10 min halftime and 20 min halftime dissociation) and slow-phase binding (approximately 1.5 h halftime and 5 h halftime dissociation) [11]. In vitro and in vivo trials have recently revealed more details on how PCSK9 mediates LDLR degradation. Recently, it has been identified that PCSK9 can also be bound to cyclase-associated protein-1 (CAP-1), which plays an important role in the LDLR degradation by PCSK9. PCSK9 binds to LDLR via its catalytic domain and interacts with CAP1 via CHRD, promoting the lysosomal degradation of protein complex LDLR/PCSK9/CAP1 by means of the caveolin pathway. Thus, the LDLR/PCSK9 complex is targeted for degradation if it depends on the caveolin-related mechanism, and for recycling if it depends on the clathrin pathway (see Figure 1). The activation of each of these pathways depends on the PCSK9 binding partner [12,13].

This conclusion is based on the fact that PCSK9-mediated LDLR degradation is suppressed in hepatic cells cultured with small interfering RNA against CAP1, as well as in heterozygous CAP1 KO murine models. Furthermore, in human PCSK9, LOF polymorphisms such as G670E or S668R demonstrate impaired interaction with CAP1 [14].

PCSK9 can act through a variety of mechanisms, which indicates that it can influence plasma lipid levels not only by modulating LDLR levels and LDL-C clearance in the liver (see Figure 1), but also by targeting other LDLR family members, including LDLR-related protein 1 (LRP1), very low-density lipoprotein (VLDL) receptor, and apoE receptor 2 (apoER2). However, the interaction of these receptors with PCSK9 does not always result in their degradation [15,16].

PCSK9 can influence lipid and lipoprotein concentrations in plasma not only through decreased lipoprotein uptake in the liver, but also by accelerating lipogenesis in the liver, which is regulated both by apoE and LDLR [17].

LDL-C plasma levels depend on the balance between hepatic triglyceride-rich VLDL expression, conversion of VLDL to LDL and LDL uptake. ApoB-100 is an important protein compound of LDL and mediates LDL binding to LDLR. Studies have shown that PCSK9 promotes hepatic and intestinal apoB-100 expression and reduces VLDLR levels in fat cells, resulting in LDLR-independent action of PCSK9 on cholesterol concentrations [18].

According to studies assessing the pleiotropic effects of PCSK9 in LDLR gene knockout murine models, expression of genes and proteins regulating lipogenesis in the liver is not affected by excessive expression of human PCSK9. However, elevated PCSK9 levels can promote the production of cholesterol and triglycerides specific to enterocytes [19]. These results indicate an LDLR-independent action of PCSK9 on the intestinal expression of triglyceride-rich lipoprotein. Thus, PCSK9 increases the secretion of triglyceride-rich lipoproteins in the intestines through mechanisms that can either involve LDLR or act independently [20].

PCSK9 is responsible for the degradation of both LDLR and structurally similar receptors, such as VLDLR and ApoER2, since all these receptors include an epidermal growth factor-like repeat A (EGF-A) domain which PCSK9 binds to and targets the complex for lysosomal degradation [21].

There have been identified over 250 mutations occurring in different PCSK9 domains [22]. Although the variations in the PCSK9 gene are responsible for only 2–4% of autosomal dominant hypercholesterolemia (ADH), they affect the plasma cholesterol levels in the general population to a much greater degree than the variation in APOB or LDLR, the latter two being the cause of 1–12% and 85–90% of familial hypercholesterolaemia (FH), respectively [23].

Although the PCSK9/LDLR interaction primarily happens through the catalytic domain of PCSK9, the majority of PCSK9 mutations in humans occur in the CHRD (n = 22; 30%), prodomain (n = 21; 28%), or the SP (n = 7; 10%) [24].

PCSK9 gene variants can be classified according to their effect on cardiovascular risk as gain-of-function (GOF) and loss-of-function (LOF) mutations. Patients with GOF mutations are susceptible to elevated LDL-C concentrations, resulting in FH and high cardiovascular risk. Studies have shown that overexpression of mutant or wild-type PCSK9 leads to a significant decrease in liver LDLR and subsequent hypercholesterolaemia. In contrast, PCSK9 KO mice demonstrate high hepatic LDLR expression and low plasma LDL-C concentrations [25].

GOF PCSK9 variations in humans, such as the Asp substitution with Tyr (PCSK9-D374Y), result in severe phenotypes with cholesterol concentrations above 500 mg/dL (~13 mmol/L) [26].

In contrast, individuals with LOF mutations in the gene present higher density of hepatic LDLR, lower LDL-C concentrations, and a decrease in lifetime cardiovascular risk (50–86% compared to individuals without the mutation). Such mutations can be observed relatively often and have been recapitulated in both in vivo and in vitro experiments [27].

A number of LOF PCSK9 polymorphisms resulting in decreased plasma PCSK9 concentrations and lower cholesterol levels can lead to impairments in secretion (S462P), trafficking (R46L), or processing (S386A) [28].

PCSK9 is also believed to affect Lp(a) levels through a mechanism involving LDLR. Studies have shown that individuals with GOF mutations in PCSK9 demonstrate elevated plasma Lp(a) levels [29].

PCSK9 is known for its involvement in lipid homeostasis but it also participates in a range of signaling pathways, including antiviral activity, apoptosis and, more recently, anti-tumor immune responses, as well as anti-oxidative housekeeping activities. Taking into account the anti-tumoral effects of anti-PCSK9 approaches and considering the wide range of therapeutic strategies of PCSK9 inhibition (monoclonal antibodies, small molecule and peptide inhibitors, antisense oligonucleotides, siRNA, etc.), it can be especially valuable as a potential therapeutic target [30].

## 3. PCSK9 in Vascular Inflammation

The pro-inflammatory effect of PCSK9 in atherosclerosis is independent of its hyperlipidemic action. Impaired clearance of ox-LDL and cholesterol efflux promotes the expression of pro-inflammatory molecules in the endothelium. Furthermore, apart from ox-LDL retention, PCSK9 can directly stimulate the release of inflammatory cytokines. Ly6C(hi) monocytes are involved in acute inflammation, their retention promoting monocytosis in the inflammatory sites. In the early stages of plaque formation, Ly6C(hi) monocytes invade the lesions and generate activated macrophages that release proinflammatory cytokines [31]. Studies in ApoE KO murine models have shown that PCSK9 increases Ly6C(hi) monocyte production in spleen and stimulates their retention in the arterial intima. Moreover, it enhances macrophage response to lipopolysaccharide, resulting in elevated expression of inflammatory cytokines such as interleukin-1β (Il-1), interleukin-6 (Il-6), and tumor necrosis factor-α (TNF-α), and a reduction in anti-inflammatory cytokines such as arginase and interleukin-10. If PCSK9 is silenced, it reduces the secretion of interleukin-1β, TNF-α, and monocyte chemoattractant protein 1 in the arteries of ApoE KO mice with atherosclerosis [32]. PCSK9 directly promotes the macrophage expression of monocyte chemoattractant protein 1 and chemokine (C-X-C motif) ligand 2 (CXCL2) that stimulate the recruitment of monocytes. These proinflammatory mechanisms involving PCSK9 are independent of cholesterol. However, they have not been detected in LDLR KO mice, indicating that the proinflammatory action of PCSK9 involves LDLRs [33].

Nuclear factor kappa B (NF-KB) is responsible for the transcription of numerous genes involved in atherosclerosis and inflammation, such as regulators of apoptosis and cell proliferation, chemokines, adhesion molecules, cytokines, and acute-phase proteins. PCSK9-mediated ox-LDL efflux depends on NF-KB, damaged mitochondrial DNA, and mitogen-activated protein kinase [34]. Elevated expression of PCSK9 in macrophages induces nuclear translocation of NF-KB, leading to higher mRNA levels of proinflammatory cytokines and toll-like receptor 4 (TLR4) release. On the other hand, inhibition of NF-KB reduces lipopolysaccharide, ox-LDL, and TNF-α-mediated expression of PCSK9. Thus, NF-KB is responsible for the signaling in the inflammatory pathway involving PCSK9 expression, and the TLR4/NF-KB pathway plays an important role in the PCSK9-induced inflammation of atherosclerotic plaques [35].

In inflammation, expression of the vascular cell adhesion molecule 1 (VCAM-1) is increased. The protein is responsible for the adhesion of basophils, eosinophils, monocytes, and lymphocytes to the artery wall. PCSK9 stimulates VCAM-1 expression in vascular SMCs, while inhibition of PCSK9 in cultured vascular SMCs leads to a substantial reduction in VCAM-1 expression [36].

Another mechanism of endothelial inflammation involves dendritic cells, which promote T-cell proliferation as well as differentiation of CD4+T cells into Th1 and Th17 in the presence of ox-LDL. Ox-LDL triggers PCSK9 secretion in the dendritic cells and promotes the expression of IL-1, IL-6, Il-10, TNF-α, and transforming growth factor beta from dendritic cells. Inhibition of PCSK9 in dendritic cells results in lower proliferation and differentiation of T-cells [37].

**Figure 2:**
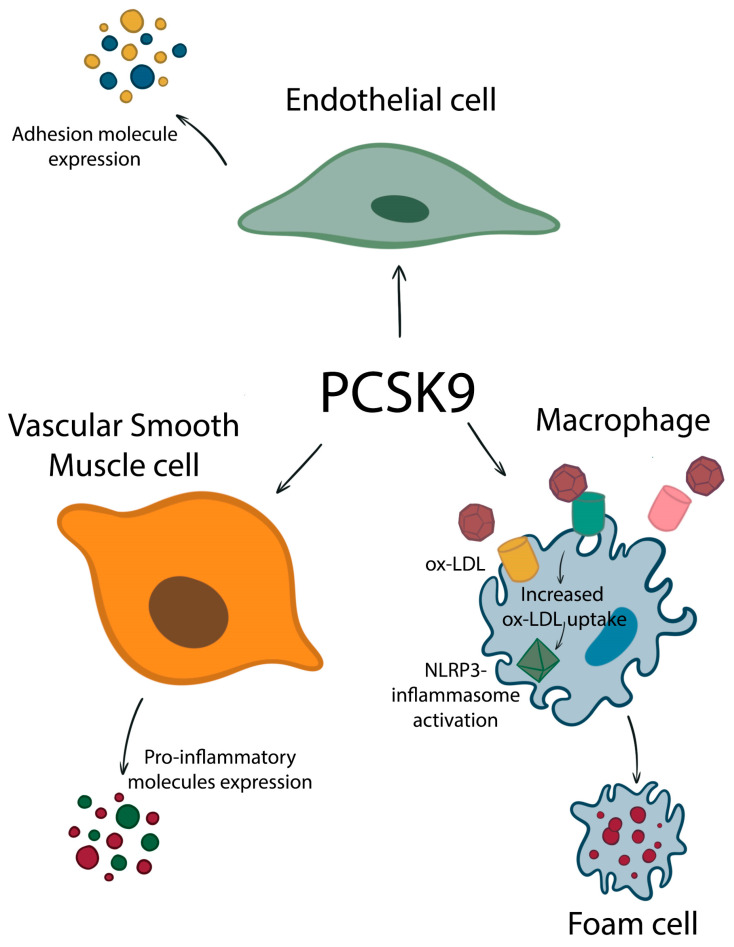
PCSK9 increases the expression of various receptors, increasing the uptake of ox-LDL. This leads to activation of NLRP3-inflammasome and increased foam-cell formation. Endothelial cells produce more adhesion molecules under the influence of PCSK9, and smooth muscle cells release more pro-inflammatory molecules.

In Figure 2, we schematize the impact of PCSK9 on the major cell types involved in atherosclerosis development.

## 4. PCSK9 in Plaque Formation

Formation of atherosclerotic plaque involves many different processes such as accumulation of lipoproteins in the arterial wall and their retention, recruitment of inflammatory cells, proliferation of vascular SMCs, matrix synthesis, apoptosis, and necrosis. There is ample evidence indicating that PCSK9 stimulates the formation of atherosclerotic plaques in mice and humans through a variety of mechanisms [38]. Firstly, it promotes LDL uptake by scavenger receptors in macrophages, accelerating the formation of foam cells; secondly, it increases apoptosis of the endothelial cells, thus reducing the vessel stability; thirdly, it contributes to the vascular inflammation. Moreover, elevated PCSK9 levels lead to lower shear stress. Thus, PCSK9 can be considered a promising target in the treatment of atherosclerotic plaques [39].

The plaque volume in the aortic root was reduced by half in murine models after anti-PCSK9 therapy. Additionally, there was observed a decrease in macrophage infiltration of the lesions as well as a decrease in serum concentrations of chemoattractants for leukocytes, such as CXCL1, CXCL3, and CXCL10, predominantly released by endothelial cells and vascular SMCs. Furthermore, PCSK9 KO mice demonstrate lower expression of VCAM-1 [40].

In addition to therapy with monoclonal antibodies, anti-PCSK9 vaccination can be used. During the vaccination, a peptide that emulates the N-terminal domain of PCSK9 is conjugated to a carrier protein that translates immunogenic properties. As a result, the immune system is activated. Long-term inhibition of PCSK9 is possible due to the generation of host-specific antibodies. Compared to monoclonal antibody therapy, vaccination has certain advantages, such as longer half-life in vitro. In addition, the doses can be administered less frequently and the total treatment cost is lower [41].

In mice, the anti-PCSK9 vaccine AT04A induces long-lasting humoral immune response against PCSK9 for 1 year, resulting in a decrease in LDL content by over 50%. Furthermore, it decreased the expression of NLRP3 inflammasome in macrophages, leading to suppressed macrophage PCSK9 secretion and slower development of atherosclerosis [42].

In individuals with acute coronary syndrome, PCSK9 inhibition used in combination with statin treatment may promote thickening of the fibrous cap, making plaque more stable. On the other hand, this effect cannot be explained solely by the LDL-C-lowering action of the treatment, which indicates that a pleiotropic effect takes place, reducing the inflammation independently of the LDL-C lowering. In atherogenic murine models, PCSK9 inhibition increased the number of circulating angiogenic cells as well as endothelial progenitor cells, which is a marker of vascular health and a predictor of decreased cardiovascular risk [43].

It turned out that PCSK9 is associated with platelet activation in the development of atherosclerosis. Li et al., in their cross-sectional study, found for the first time that there was a positive and independent association between plasma PCSK9 levels and platelet counts in 330 patients with stable CAD, suggesting an association between high PCSK9 levels, platelets, atherosclerosis, and cardiovascular disease [44]. Another study by Pastori et al. showed a strong relationship between elevated PCSK9 levels and high urinary excretion of 11-dehydrothromboxane B2 (11-dh-TxB2), a stable metabolite of thromboxane A2. The study was conducted on patients with a high risk of cardiovascular complications [45]. Thus, urinary excretion of 11-dh-TxB2 is widely used as a predictive marker of myocardial infarction or cardiovascular disease in patients treated with aspirin. A potential mechanism underlying the association between urinary 11-dh-TxB2 and PCSK9 could lead to the possible involvement of cyclooxygenase (COX)-1, an important enzyme for thromboxane A2; however, other mechanisms should also be taken into account [46].

## 5. Current PCSK9 Inhibitors

In spite of the fact that statin therapy is the most common treatment to reduce circulating LDL-C and cholesterol in subjects with high risk for cardiovascular disease, more than 70 per cent of patients receiving statin monotherapy do not demonstrate LDL-C levels lower than 70 mg/dL. Furthermore, many patients discontinue statin therapy within a year due to the necessity of daily drug administration, and adverse effects such as myalgias and myopathies. At the same time, poor therapy adherence is a significant factor increasing the risk of CVD [47].

Consequently, the pharmaceutical industry has had to develop ways to improve patient adherence to existing therapies and look for new LDL-C-lowering therapies that would be well-tolerated. As soon as PCSK9′s role in cholesterol homeostasis had been discovered, there appeared interest to create therapies targeting its pathway which could be used in hypercholesterolemia treatment [48]. It is important to note that inhibition of PCSK9 with evolocumab, alirocumab, and inclisiran is currently used as the third line of hypolipemic treatment, recommended after administration of the maximum tolerated dose of statin and ezetimibe.

Despite statins being the primary line treatment prescribed for LDL-C lowering, linear dose-dependent lowering was not observed. Moreover, the “statin escape phenomenon” was also described, according to which LDL-C levels are reported to increase following prolonged statin therapy. Statins lower hepatic intracellular cholesterol, which leads to an enhancement of sterol-regulatory element binding protein-2 (SREBP-2) nuclear translocation. This activates LDLRs and PCSK9 gene expression, and increases circulating PCSK9 levels. Both PCSK9 and LDLR levels are increased by statin therapy. Increased PCSK9 binds to LDLR and leads to lysosomal degradation instead of regular recycling. This is a potential mechanism of limitation of the efficacy of statin-induced LDL-C reduction, which can explain the “6% rule for statins”, which states that the doubling of a statin dose brings only approximately 6% additional reduction in LDL-C level [49,50].

We summarize the basic information about trials on three currently used PCSK9 inhibitors in Table 1, and discuss their results in the text below.

Since the major part of LDL receptors is reused while PCSK9 prevents their recycling in cells, a monoclonal antibody block would promote LDLR recycling on cell membranes and induce LDL-C uptake from plasma and a decrease in circulating LDL-C [55].

Currently, two monoclonal antibodies, evolocumab and alirocumab, have been developed using transgenic mouse models. They prevent EGF-A and PCSK9 interaction on LDLR and were included as additional drugs in the standard of care for clinical hypercholesterolemia treatment of patients with evident CVD with or without familial hypercholesterolemia. These mAbs markedly, up to 60 to 70 per cent, lower circulating LDL-C, which significantly reduces the risks of cardiovascular disease [56].

There are currently two approved schemes of evolocumab administration: an injection of 140 mg every two weeks, or an injection of 420 mg every four weeks. Both schemes resulted in almost the same decrease in LDL-C levels. Moreover, these doses sustained maximal LDL-C reduction, resulting in greater stability in LDL-C reduction over the dosing interval compared to lower doses. In Phase I studies, pharmacokinetics of evolocumab at a dose range of 21–420 mg appeared to be non-linear, which means that the drug concentration in plasma does not increase strictly proportionally to the administered dose. Clearance can be described by parallel linear and non-linear clearance pathways: evolocumab pK reached linearity at single doses > 210 mg injected subcutaneously with tmax at 72 h. Repeated doses over 140 mg exhibited linear kinetics. Again, in Phase II studies, AMG145 kinetics followed approximately linear profile at doses > 140 mg every 2 weeks, both when administered alone or with statins [57]. Similar results were described for another inhibitor, alirocumab [58,59].

PCSK9 inhibitors appeared to decrease Lp(a) levels, which are not affected by statins. Various mechanisms were proposed to implement such an effect. According to one of the hypotheses, Lp(a) competes mildly with LDL for LDLR binding. Therefore, when PCSK9 inhibitors enhance LDLR expression and LDL levels are low, Lp(a) levels lower. According to other versions, it is implemented via decreased apoB or Lp(a) synthesis.

Thus, the reduction in Lp(a) levels achieved was 21.7–24.7% by evolocumab [60], 13–29% by alirocumab [61,62], and 18.2–125.6% by inclisiran [63].

In 2013–2015, the FOURIER double-blind, placebo-controlled trial was organized, for which 27,564 multinational patients from 49 countries were randomized at 1242 sites [51]. The trial tested safety and clinical efficacy of evolocumab used together with statin therapy of high or moderate intensity in patients with established atherosclerotic cardiovascular disease. The median follow-up of 26 months showed that evolocumab (140 mg biweekly or 420 mg monthly) reduced LDL-C levels by 60 per cent from baseline levels, i.e., to 30 mg/dL from a median of 92 mg/dL. Moreover, evolocumab therapy led to a 15% reduction in the primary efficacy endpoint, or major cardiovascular events, and proved beneficial for patients with prior ischemic stroke, metabolic syndrome, and diabetes. Noteworthily, only 0.3 per cent of patients from the whole trial population produced evolocumab-binding antibodies without developing neutralizing antibodies.

In 2012 to 2015, the ODYSSEY OUTCOMES multicenter, double-blind, placebo-controlled study was carried out, for which 18,924 patients from 57 countries were randomized at 1315 sites [52]. The trial tested safety and clinical efficacy of alirocumab at a dose of 75 to 150 mg biweekly used together with statin therapy of high or moderate intensity (maximum toleration) in patients who had a previous coronary syndrome and high atherogenic lipoprotein levels in spite of statin treatment. The median follow-up of 32 months showed that alirocumab was superior to placebo in reducing LDL-C by more than 50% and reducing MACE by 15% (including CHD death, fatal/nonfatal stroke, unstable angina pectoris, and non-fatal MI that required hospitalization).

These trials also demonstrated that patients with a higher risk score showed a larger risk reduction, in absolute as well as relative terms, and both drugs reduced the venous thromboembolism risk by 31%. The trials proved that inhibitors of PCSK9 can be used as adjunctive therapy in dyslipidemia treatment. This was included in the ACC guidelines for clinical practice. According to them, inhibitors of PCSK9 are recommended for patients with clinically evident atherosclerotic cardiovascular disease and LDL-C ≥ 90 mg/dL in spite of statin treatment in maximal tolerated doses with or without ezetimibe [64].

There are concerns, however, about whether patients would be compliant with the necessity to receive a subcutaneous injection every 2 weeks or every month. A small-scale, real-world evidence trial revealed that compliance with PCSK9 monoclonal antibodies was better than that with statins. This can be, at least partially, explained by the fact that the patients felt reassured due to substantial LDL-C reduction to recommended LDL-C levels even if the levels had been very high previously [65]. Only about 12 percent of patients were partially compliant and 9 per cent noncompliant with the regimen, which proved that it is easier to adhere to a biweekly or a monthly procedure, than to daily drug administration. However, the data indicate that 30–40% of patients stopped using their anti-PCSK9 mAb therapy during/after 6 months of treatment initiation. Moreover, a current in-depth analysis of data on real-world adherence to and persistence with LLT in Germany demonstrated a rate of treatment discontinuation of 50% after 36 months [66,67].

The knowledge that less-frequent drug administration increases patients’ compliance inspired new investigations of therapies inhibiting PCSK9 production in cells. Thus, inclisiran (inclisiran sodium) was developed, a chemically synthesized small interfering RNA molecule that acts as a PCSK9 inhibitor. Significantly reducing PCSK9 production in the liver, it promotes marked LDL-C reduction, preserving its therapeutic effects for six months after a subcutaneous injection [68]. Therefore, it can be administered twice a year, which increases patients’ compliance. At the same time, it provides substantial LDL-C reduction (over 50%), which can be compared to that of statins of high intensity. This is why inclisiran can be used for CVD prevention [69].

Twenty-four hours after the injection, inclisiran cannot be detected in plasma because it is rapidly and specifically uptaken by liver cells. In spite of PCSK9 presence in tissues outside the liver, inclisiran specifically targets hepatic PCSK9 without interference with PCSK9 synthesis in non-hepatic tissues [70].

The drug’s mechanism of action is based on intracellular binding of inclisiran guide strand to an RNA-induced silencing complex, which hybridizes specifically to the PCSK9-encoding mRNA molecules, resulting in mRNA cleavage and its catalytic degradation. This prevents hepatic PCSK9 synthesis. Thus, several PCSK9 mRNAs can be degraded by a single inclisiran–RISC complex [71].

ORION trials have been studying inclisiran safety and efficacy in reducing LDL-C [72]. The third phase, which is in progress now, is assessing its long-term effectiveness and safety as well as efficacy in homozygous FH patients.

ORION 9, 10, and 11 trials have proved inclisiran 284 mg has good tolerability and efficacy in long-term LDL-C reduction; PCSK9 plasma levels were reduced by about 80% in patients with a very high risk of cardiovascular disease [53].

ORION 4 and 5 studies have been evaluating the impact of inclisiran on CV outcomes in adult patients with evident ASCVD and in adult homozygous FH patients, respectively.

Pivotal Phase III studies confirmed that inclisiran can be used as a therapy for primary hypercholesterolemia (both familial and non-familial heterozygous hypercholesterolemia). Since December 2020, it has been approved for use in the EU [73].

Another strategy aimed at inhibiting synthesis of PCSK9 may use base editors that generate precise single-nucleotide changes. In general, new technologies based on gene editing are a potential for clinical applications in the future, including, in particular, PCSK9 targeting for hypercholesterolemia management [54].

Natural compounds also include those that can inhibit transcription and translation of PCSK9. One such substance is berberine. It can be found in the roots and stems of a number of plants, in particular the Berberis species, as well as the rhizomes of Koptis and Hydratis Canadensis. In addition to these properties, berberine is able to stimulate LDLR transcription and translation in a PCSK9-independent manner.

Lupine has a similar effect which reduces circulating PCSK9 levels and inhibits PCSK9 binding to LDLR. Moreover, polyphenols present in many plant foods (fruits, vegetables, nuts, red wine, etc.) are also involved in the suppression of the PCSK9 protein. One such polyphenolic compound, resveratrol, suppresses PCSK9 expression by inhibiting the SREBP-1 pathway. Another polyphenol, quercetin, on the contrary, is able to activate SREBP2 transcription, increasing the expression of the LDLR gene and reducing the expression of PCSK9 [74].

In addition to their cardiovascular efficacy, PCSK9 inhibitors have other potential applications. According to recent studies, PCSK9 inhibitors may be useful in the treatment of sepsis, certain tumors, certain viral infections, and other diseases [75].

The small non-randomized trials conducted by Basiak et al. revealed that PCSK9 can potentially impact hemostatic variables in subjects with isolated hypercholesterolemia. Many studies have shown an important role of PCSK9 in the pathogenesis of cardiovascular diseases, partly independent of its effect on lipid metabolism. The data obtained by Basiak et al. also suggest that PCSK9 may influence primary and secondary hemostasis either indirectly, through its effect on LDL-C, or directly, through its effect on platelet activation and plasma FVIII levels. In addition, therapy can be effective even in mild lipid disorders and in patients with statin intolerance or contraindications to their use. Unfortunately, the use of PCSK9 inhibitors is very expensive, so in clinical practice it is used only for selected patients, patients with high or very high cardiovascular risk, and in patients with statin intolerance [76].

## 6. Conclusions

In this review, we have looked into mechanisms through which PCSK9 can contribute to the development of atherosclerosis. Firstly, PCSK9 affects lipid metabolism in several ways, some of which are due to its interaction with LDLR. Studies have shown that PCSK9 can promote lipogenesis and at the same time decrease lipoprotein uptake through various pathways, leading to higher plasma lipid levels. Secondly, PCSK9 stimulates vascular inflammation, and this effect is independent of its ability to increase lipid levels in plasma. Several therapies targeting PCSK9 have been developed, including monoclonal antibody therapy, anti-PCSK9 vaccination, and RNA interference therapy. In addition to a long-lasting lipid-lowering effect, these therapies require less-frequent administration compared to standard statin treatment. Thus, maintaining the regimen becomes much easier for patients. New technologies involving gene-editing provide basis for the development of completely new drugs targeting PCSK9. Therefore, this approach definitely deserves more attention as an object of further research in the field of CVD treatment.

## Figures and Tables

**Table 1:** Trials on the efficacy and safety of PCSK9 inhibitors.

Trial	Drug	Dosage	Subjects	LDL-C	Reference
FOURIER	Evolocumab (with statins)	140 mg biweekly or 420 mg monthly	27,564 ASCVD patients	Reduction by 60%	[51]
ODYSSEY OUTCOMES	Alirocumab (with statins)	75 to 150 mg biweekly	18,924 ASCVD patients	Reduction by more than 50%	[52]
ORION 9	Inclisiran	284 mg on day 1, day 90, and 6-monthly thereafter	482 heterozygous familial hypercholesterolaemia patients	Reduction by 53.8%	[53]
ORION 10	Inclisiran	284 mg on day 1, day 90, and 6-monthly thereafter	1561 ASCVD and elevated LDL-C patients	Reduction by 47.2%	[53]
ORION 11	Inclisiran	284 mg on day 1, day 90, and 6-monthly thereafter	1617 ASCVD or risk equivalents and elevated LDL-C patients	Reduction by 47.9%	[53]
ORION 4	Inclisiran	300 milligram (mg) inclisiran sodium for injection administered by SC injection on day 1 and day 90	15,000 ASCV patients	N/A	[54]
